# Leptin down-regulates KCC2 activity and controls chloride homeostasis in the neonatal rat hippocampus

**DOI:** 10.1186/s13041-020-00689-z

**Published:** 2020-11-12

**Authors:** Camille Dumon, Yasmine Belaidouni, Diabe Diabira, Suzanne M. Appleyard, Gary A. Wayman, Jean-Luc Gaiarsa

**Affiliations:** 1 Aix-Marseille Univ UMR 1249, INSERM (Institut National de La Santé et de La Recherche Médicale) Unité 1249, INMED (Institut de Neurobiologie de La Méditerranée), Parc Scientifique de Luminy, Marseille, France; 2 Program in Neuroscience, Department of Integrative Physiology and Neuroscience, Washington State University, Pullman, WA USA; 3 Present Address: Neurochlore Parc Scientifique et Technologique de Luminy, Bâtiment Beret Delaage, Zone Luminy Entreprises Biotech, Marseille, France

**Keywords:** GABA, KCC2, Chloride homeostasis, Hippocampus, Rat, Leptin, Maternal obesity

## Abstract

The canonical physiological role of leptin is to regulate hunger and satiety acting on specific hypothalamic nuclei. Beyond this key metabolic function; leptin also regulates many aspects of development and functioning of neuronal hippocampal networks throughout life. Here we show that leptin controls chloride homeostasis in the developing rat hippocampus in vitro. The effect of leptin relies on the down-regulation of the potassium/chloride extruder KCC2 activity and is present during a restricted period of postnatal development. This study confirms and extends the role of leptin in the ontogenesis of functional GABAergic inhibition and helps understanding how abnormal levels of leptin may contribute to neurological disorders.

## Introduction

Leptin, the product of the obese (ob) gene, is a circulating hormone secreted mainly from the white adipocytes and transported across the blood brain barrier to the hypothalamus to suppress appetite and enhance metabolism in adult [1]. The hypothalamus is not the only central nervous system target for leptin, as a high density of leptin receptors are expressed in other brain areas including the hippocampus where leptin receptors regulate many aspects of synaptic plasticity and cognitive function [2, 3]. A large body of evidence indicates that leptin also acts as an important neurodevelopmental factor during the perinatal period [4–6]. Thus, while plasma leptin levels reflect adiposity in adult rodents, leptin levels surge during the two first postnatal weeks of life regardless of the animal’s weight or body fat mass [1]. A similar restricted surge of plasma levels is observed during the last trimester of gestation in human [7]. Along with the leptin surge, leptin receptors are expressed and functional in several brain regions at embryonic and postnatal stages and activation of these receptors promote neuronal networks development [8–14]. Due to the many important physiological and developmental functions of leptin, dysregulation in its availability or signaling has been proposed as causal factors for the occurrence of neurological disorders [15–23].

Abnormalities in GABAergic synaptic transmission are strongly associated with neurological disorders [24, 25]. Therefore, understanding whether and how leptin controls the development and efficacy of the GABAergic transmission is warranted. Leptin deficient (*ob/ob*) mice exhibit a lower number of GABAergic synapses impinging hypothalamic [26] and hippocampal [12] neurons highlighting the role of leptin in GABAergic synaptogenesis. Likewise, leptin modulates the GABAergic synaptic activity in vitro in the hypothalamus [27, 28] and hippocampus [12, 29]. The homeostasis of intra-neuronal Cl^−^ concentration ([Cl^−^]_i_) is an essential determinant of GABA functioning and alterations in [Cl^−^]_i_ is implicated in the etiology of numerous neurological and psychiatric disorders [30–32]. In a previous study, we reported that the absence of leptin signaling accelerates the ontogenesis of functional GABAergic inhibition in the newborn mice hippocampus in vivo [13]. In the present study we show that leptin acts directly on hippocampal neurons to control Cl^−^ homeostasis and the activity of the K^+^-Cl^−^ co-transporter KCC2 in the rat hippocampus during a restricted developmental window.

## Materials and methods

All animal procedures were carried out in accordance with the European Union Directive of 22 September (2010/63/EU). The protocol was approved by the INSERM Local committee (Number 0287.01, delivered by the French Ministry of Education and Research). Experiments were performed on both male and female postnatal day (P) 1 to 10 Wistar rats. Animals were housed in a temperature-controlled environment with a 12 light/dark cycle and free access to food and water.

### Hippocampal slice preparation

Brains were removed and immersed into ice-cold (2–4 °C) artificial cerebrospinal fluid (ACSF) with the following composition (in mM): 126 NaCl, 3.5 KCl, 2 CaCl_2_, 1.3 MgCl_2_, 1.2 NaH_2_PO_4_, 25 NaHCO_3_ and 11 glucose, pH 7.4 equilibrated with 95% O_2_ and 5% CO_2_. Hippocampal slices (600 µm thick) were cut with a McIlwain tissue chopper (Campden Instruments Ltd.) and kept in ACSF at room temperature (25 °C) for at least one hour before recording. Slices were then transferred to a submerged recording chamber perfused with oxygenated (95% O_2_ and 5% CO_2_) ACSF (3 ml/min) at 34 °C.

### Electrophysiological recordings

Perforated patch-clamp recordings were made from CA3 pyramidal neurons. The pipettes (4–7 MΩ) were tip filled with an internal solution of 150 mM KCl and 10 mM HEPES, (pH adjusted to 7.2 with Tris-OH) and then backfilled with the same solution containing gramicidin A (50 µg/ml, diluted from a stock solution of 50 mg/ml in DMSO). Data were acquired with an axopatch 200B amplifier (Molecular Devices LLC, San Jose, USA). A stimulating bipolar tungsten electrode was placed in the CA3 *stratum radiatum* to evoke GABA_A_ receptor-mediated postsynaptic currents (eGABA_A_-PSCs) at a frequency of 0.01 Hz in the presence of glutamatergic receptor antagonists (NBQX 5 µM and D-APV 40 µM). After the access resistance had dropped to 40–80 MΩ and stabilized (15–30 min), we varied the starting holding potential (− 70 mV) in increasing and decreasing steps of 10 mV and measured the peak amplitude of averaged eGABA_A_-PSCs (3 single sweeps) to construct a current–voltage relationship. Leptin was applied for 20 min and a second current–voltage relationship was conducted. Measurements were not corrected for the liquid junction potentials. A linear regression was used to calculate the best-fit line of the voltage dependence of the synaptic currents. Spontaneous rupture into whole-cell was evidenced by large inward synaptic currents due to E_Cl_ of 0 mV.

Loose cell attached patch clamp recordings of action potential firing were performed from CA3 pyramidal neurons in the voltage-clamp mode at pipette potential of 0 mV using an axopatch 200B (Molecular Devices LLC, San Jose, USA). The glass electrodes (4–7 MΩ) filled with an internal solution of 150 mM KCl and 10 mM HEPES (pH adjusted to 7.2 with Tris–OH). After a baseline period of at least 10 min in the presence of NBQX (5 µM) and D-APV (40 µM), Leptin was bath applied for 20 min. The effect of leptin was quantified as the mean frequency of action potential at the end of the leptin application (15–20 min) versus baseline frequency (− 10–0 min).

Evoked synaptic activity and spontaneous action potentials were recorded with Axoscope software version 8.1 (Molecular Devices LLC, San Jose, USA) and analyzed offline with Mini Analysis Program version 6.0 (Synaptosoft).

### Drugs

The following reagents were purchased from the indicated sources: 1,2,3,4-tetrahydro-6-nitro-2,3-dioxo-benzo [f]quinoxaline-7-sulfonamide (NBQX) and D-2-amino-5-phospho-valeric acid (D-APV) from the Molecular, Cellular, and Genomic Neuroscience Research Branch (MCGNRB) of the National Institute of Mental Health (NIMH, Bethesda, MD, USA). Leptin and VU0463271from Tocris Cookson (Bristol, UK). Bumetanide and Gabazine from Sigma (St Louis, MN, USA).

### Statistics

Statistical analyses were conducted with GraphPad Prism (GraphPad software 5.01). Shapiro–Wilk normality test was used to determine the normality of distributions. *P* < 0.05 was considered significant for this and all subsequent tests. Our data displaying non-normal distribution, we used a Two-tailed Mann–Whitney *U* test for comparison between two independent groups, and a Two-tailed Wilcoxon matched-pairs signed rank test to compare paired data. To ensure the consistency and reproducibility of our results, we conducted repeated trials in different acute hippocampal slices prepared from at least three different animals for each experimental condition. All data are expressed as mean ± standard error to the mean (S.E.M.). In the figures, box plots represent the 1st and 3rd quartiles; whiskers show data range; horizontal lines show the median.

## Results

### Leptin controls chloride homeostasis in vitro

**Fig. 1:**
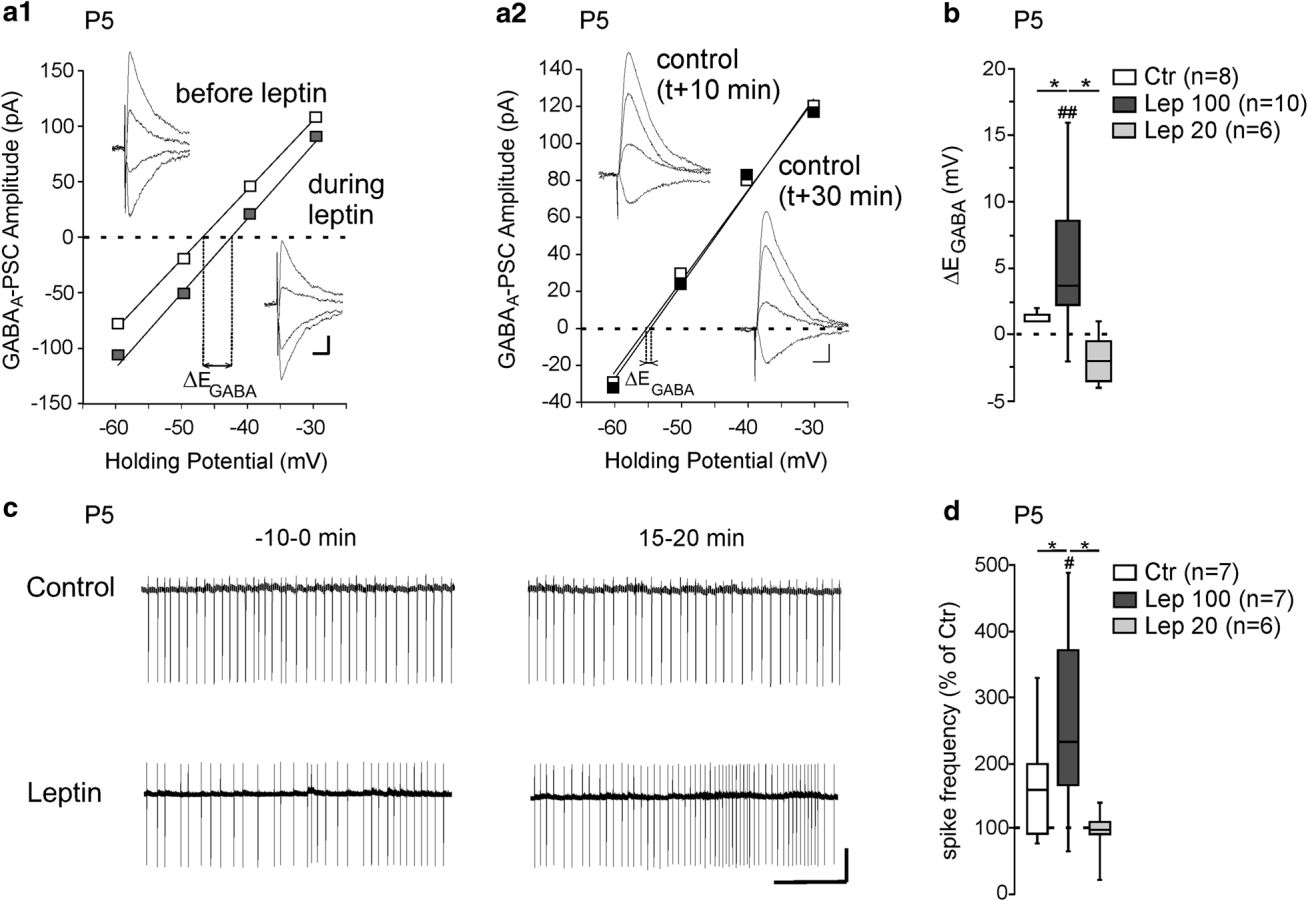
Leptin controls chloride homeostasis in rat hippocampal slices. **a** Current–voltage (I–V) relationships for evoked GABAergic synaptic currents before and during leptin application (100 nM, 20 min) **a1** and in control experiments **a2** during which neurons were recorded following the same protocol but leptin was omitted. The intercepts of the linear regression of the I–V curves was used to calculate E_GABA_ changes induced by leptin (∆E_GABA_). Insets depict the GABAergic synaptic currents. Scale bars, 10 ms, 20 pA. **b** Box plots of ∆E_GABA_ induced by leptin 100 and 20 nM applied during 20 min. In control experiments (Ctr), neurons were recorded following the same protocol in the absence of leptin. **c** Loose patch recordings of CA3 pyramidal neurons on acute hippocampal slices before (− 10–0 min) and during (15–20 min) the application of leptin (100 nM, 20 min) and in control experiment, during which neurons recorded following the same protocol in the absence of leptin. Scale bar, 2 min, 50pA. **d** Box plots of leptin action on spike activity in the indicated conditions. ^##^P < 0.01 when compared to pre-leptin values, two-tailed Wilcoxon paired test. *P < 0.05 when compared to leptin experiments, two-tailed Mann Whitney test

Our first aim was to determine whether leptin directly acts on hippocampal cells to control Cl^−^ homeostasis in the neonatal rat. We used acute postnatal (P) day 5 rat hippocampal slices and stimulated presynaptic GABAergic neurons while gramicidin perforated patch-clamp recordings were made from CA3 pyramidal neurons in the presence of the glutamatergic receptor blockers NBQX (5 µM) and D-APV (40 µM). GABA_A_ receptor-mediated postsynaptic currents (eGABA_A_-PSCs) were evoked at different holding potentials, before and during the application of leptin (100 nM, 20 min), to determine the impact of the adipocyte hormone on their reversal potential (E_GABA_). We found that leptin induced an average depolarizing shift of E_*G*ABA_ (∆E_GABA_) of 5.4 ± 1.7 mV (from − 48.2 ± 2.8 to − 42.8 ± 3.7 mV, n = 10, z = − 2.5, *p* = 0.005, Fig. 1a1, b). In control experiments in which leptin was omitted E_GABA_ did not change over the same recording duration (from − 45.6 ± 3.8 to − 45.2 ± 3.8 mV, n = 8, z = − 1.2, *p* = 0.21, ∆E_GABA_ = 1.3 ± 0.5 mV, U = 16.5, *p* = 0.03 vs leptin 100 nM response, Fig. 1a2, b). Leptin applied at a concentration of 20 nM for 20 min had no effect on E_GABA_ (from − 53.6 ± 2.4 to − 54.8 ± 3.1 mV, n = 6, z = − 1.5, *p* = 0.5, ∆E_GABA_ = − 0.5 ± 1.6 mV, U = 11, *p* = 0.09 vs control experiment, Fig. 1b). We next determined whether the depolarizing shift of E_GABA_ induced by leptin was associated with increased neuronal excitation. To this end we recorded action potentials in loose patch mode in the presence of NBQX (5 µM) and D-APV (40 µM). After a baseline period (10 min), leptin (100 nM) was added to the perfusion medium for 20 min. We assessed the effect of leptin on action potential firing at the end of the leptin application (15–20 min) versus the baseline period (− 10–0 min, Fig. 1c). Leptin led to a significant increase in the frequency of action potentials (from 0.46 ± 0.14 Hz to 1.02 ± 0.32 Hz, n = 7, z = − 2.1, *p* = 0.03, Fig. 1c, d). In interleaved control experiments in which leptin was omitted the spike firing remained constant (from 0.32 ± 0.12 to 0.43 ± 0.16 Hz, n = 7, z = − 1.1, *p* = 0.29 vs baseline and U = 16, *p* = 0.04 vs leptin 100 nM response, Fig. 1c, d). In agreement with the lack of effect of leptin at 20 nM on E_GABA_ (Fig. 1b), bath applied leptin at the same concentration (20 nM, 20 min) had no effect on the firing frequency of CA3 pyramidal neurons (from 0.47 ± 0.14 to 0.44 ± 0.16 Hz, n = 6, z = − 0.5, *p* = 0.68 vs baseline and U = 17, *p* = 0.12 vs control experiment, Fig. 1b). Altogether these data show that bath applied leptin shifts E_GABA_ towards depolarizing values and increases the neuronal excitation of P5 CA3 pyramidal neurons on rat hippocampal slices.

### Leptin controls KCC2 activity in vitro

**Fig. 2:**
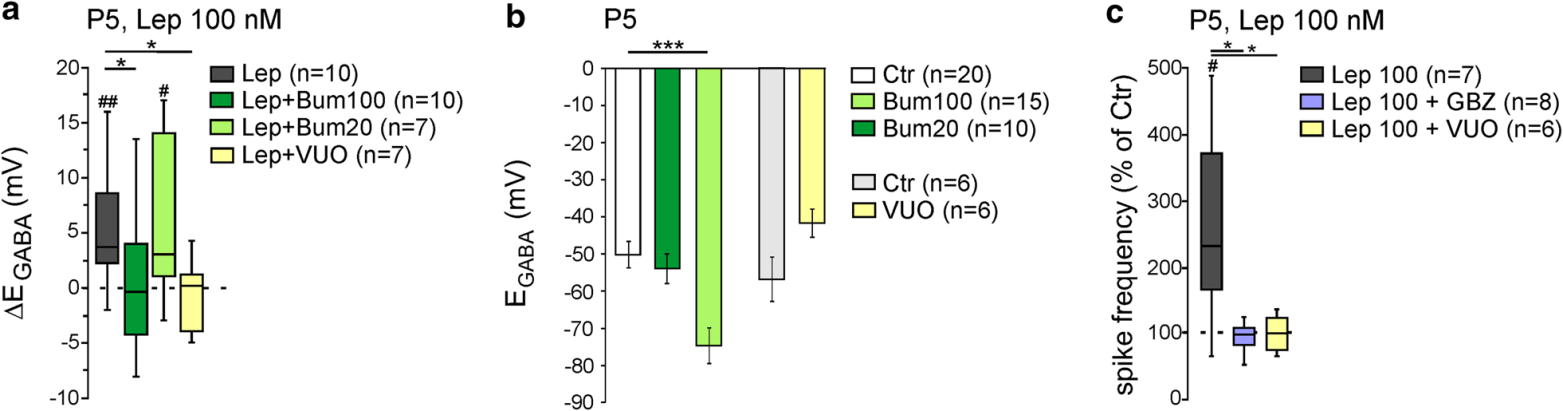
Leptin controls KCC2 activity in rat hippocampal slices. **a** Box plots of ∆E_GABA_ induced by leptin 100 nM (20 min) in control condition (Lep), in the presence of bumetanide 100 and 20 µM, or in the presence of VU0423271 (VU0, 10 µM). **b** Bar plots of the mean and standard error to the mean of the reversal potential of GABA_A_ receptor-mediated postsynaptic currents (E_GABA_) in the indicated conditions. **c** Box plots of leptin action (100 nm, 20 min) on spike activity in the presence of Gabazine (GBZ, 5 µM), or in the presence of VU0423271 (VU0, 10 µM). ^#^P < 0.05 and ^###^P < 0.01 when compared to pre-leptin values, two-tailed Wilcoxon paired test. *P < 0.05 and ***P < 0.001 when compared to leptin experiments, two-tailed Mann Whitney test

Chloride homeostasis and the strength of GABA_A_-mediated synaptic inhibition are mainly controlled by the activity of two cation-chloride cotransporters: the Na^+^-K^+^-2Cl^−^ (NKCC1) co-transporter that accumulates Cl^−^ intracellularly and the K^+^-Cl^−^ (KCC2) co-transporter that lowers intracellular Cl^−^ concentration [33, 34]. We therefore asked whether leptin acts on KCC2 and/or NKCC1 activity. We found that the diuretic bumetanide at a concentration of 100 µM, to block both NKCC1 and KCC2 had no effect on E_GABA_ (from − 51.4 ± 2.9 (n = 20) to − 55.6 ± 4.2 mV, (n = 15), U = 135, *p* = 0.6, Fig. 2b) but prevented the depolarizing shift of E_GABA_ induced by leptin (100 nM, 20 min) (from − 54.7 ± 4.4 to − 54.6 ± 5.7 mV, n = 10, z = − 0.02, *p* = 0.85, ∆E_GABA_ = − 0.1 ± 2.0 mV, U = 22.5, *p* = 0.03 vs leptin 100 nM response, Fig. 2a). However, bumetanide at 20 µM to block NKCC1 shifted E_GABA_ toward hyperpolarizing values (from − 51.4 ± 2.9 (n = 20) to − 75.4 ± 4.4 mV (n = 10), U = 23.5, *p* = 0.009, Fig. 2b) but failed to prevent the effect of leptin (100 nM, 20 min) on E_GABA_ (from − 75.4 ± 5.8 to − 67.4 ± 6.2 mV, n = 7, z = − 2.1, *p* = 0.04, ∆E_GABA_ = 8.2 ± 2.9 mV, U = 28, *p* = 0.51 vs leptin 100 nM response, Fig. 2a). These results suggest that leptin down-regulates KCC2 activity. Accordingly, the selective KCC2 blocker VU0463271 (20 µM) led to a non-significant depolarizing shift of E_GABA_ (from − 57.5 ± 6.1 to − 42.3 ± 4.0 mV, n = 6, z = − 1.9, *p* = 0.06, Fig. 2b) and prevented the effect of leptin (100 nM, 20 min) (from − 43.8 ± 3.2 to − 44.7 ± 3.8 mV, n = 7, z = − 0.6, *P* = 0.65, ∆E_GABA_ = − 0.8 ± 1.2 mV, U = 9.5, *p* = 0.01 vs leptin 100 nM response, Fig. 2a).

To determine whether the increase in spike firing induced by bath applied leptin (Fig. 1c, d) also resulted from a down regulation of KCC2 activity and a modification of GABAergic strength, the same experiment was repeated in the continuous presence of the selective GABA_A_ receptor antagonist Gabazine (5 µM) or in the presence of the selective KCC2 blocker VU0463271. We found that Gabazine (5 µM) completely abolished the leptin-induced (100 nM, 20 min) increase in firing. The frequency of action potential was respectively 0.81 ± 0.22 Hz and 0.85 ± 0.28 Hz before and during the application of leptin (n = 8, z = − 0.07, *p* = 0.96 vs baseline and U = 7, *p* = 0.01 vs leptin 100 nM response, Fig. 2c). Likewise, the selective KCC2 blocker VU0463271 (20 µM) also prevented the effect of leptin (100 nM, 20 min) (from 0.23 ± 0.06 to 0.21 ± 0.04 Hz before and during the application of leptin, n = 6, z = − 0.4, *p* = 0.72 vs baseline and U = 6, *p* = 0.03 vs leptin 100 nM response, Fig. 2c). Altogether, these data show that leptin down-regulates KCC2 activity shifting E_GABA_ towards depolarizing values in P5 rat hippocampal slices.

### The action of leptin in vitro on chloride homeostasis is developmentally regulated

**Fig. 3:**
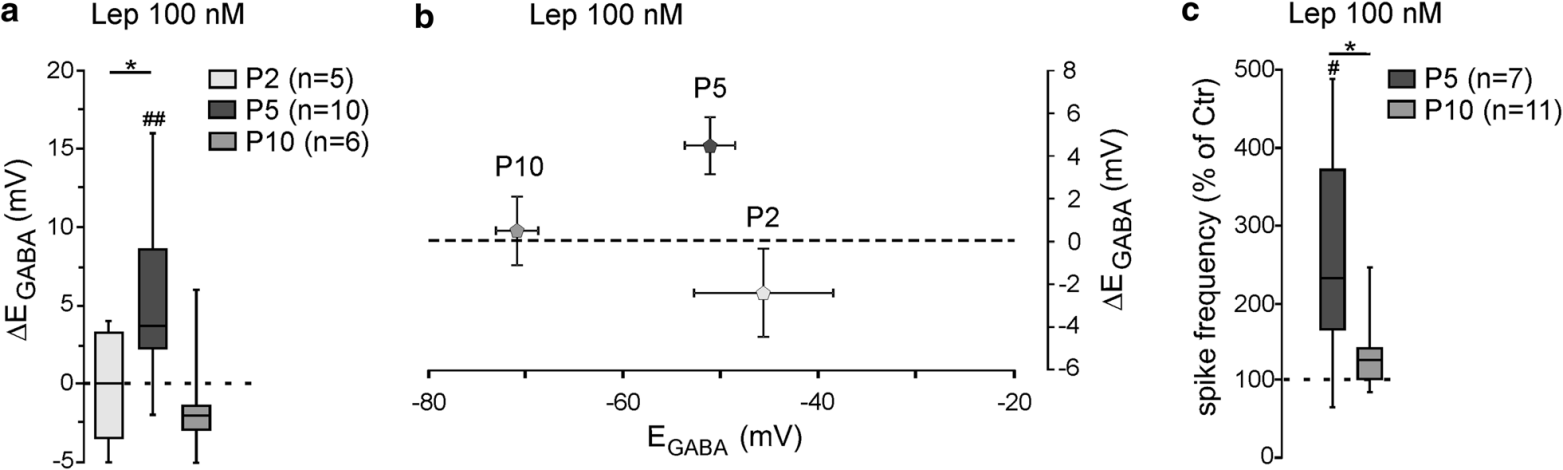
The action of leptin of chloride homeostasis is developmentally regulated. **a** Box plots of ∆E_GABA_ induced by leptin (100 nM, 20 min) at postnatal (P) day 2, 5 and 10. **b** Plots of mean and standard error to the mean of the ∆E_GABA_ induced by leptin (100 nM, 20 min) versus the reversal potential of GABA_A_ receptor-mediated postsynaptic currents (E_GABA_) at P2, P5 and P10. **c** Box plots of leptin action (100 nM, 20 min) on spike activity at P5 and P10. ^#^P < 0.05 when compared to pre-leptin values, two-tailed Wilcoxon paired test. *P < 0.05 when compared to leptin response at P5, two-tailed Mann Whitney test

Previous studies reported that the responsiveness of leptin is regulated during development [29, 35–37]. We therefore asked whether the leptin-induced depolarizing shift of E_GABA_ is developmentally regulated. We found a non-linear bell-shaped relationship between the age of the rats and the responsiveness of leptin. Thus, while bath applied leptin (100 nM, 20 min) led to a significant depolarizing shift of E_GABA_ at P5 (Fig. 1b), the same application had no effect on the reversal potential of GABA_A_-PSCs evoked on hippocampal slices at P2 (from − 45.6 ± 7.1 to − 47.6 ± 6.4 mV, n = 5, z = − 0.9, *p* = 0.43, ∆E_GABA_ = − 0.7 ± 2.1 mV, *U* = 7, *P* = 0.02 vs leptin 100 nM response at P5, Fig. 3a) and P10 (from − 70.8 ± 2.1 to − 70.3 ± 2.7 mV, n = 6, z = − 0.1, *p* = 0.99, ∆E_GABA_ = 0.5 ± 1.6 mV, U = 14, *p* = 0.08 vs leptin 100 nM response at P5, Fig. 3a). Of note, the effect of leptin on E_GABA_ was not correlated to the initial polarity of the GABAergic responses (Fig. 3b). Likewise, leptin (100 nM, 20 min) failed to increase the firing frequency of CA3 pyramidal neurons when applied at P10 (from 0.55 ± 0.13 to 0.64 ± 0.13 Hz before and during the application of leptin, n = 11, z = − 1.8, *p* = 0.1 and U = 16, *p* = 0.9 vs baseline leptin 100 nM response at P5, Fig. 3c). We were unable to test the effect of leptin at P2 because of a sparse action potentials and low frequency discharge. Altogether, these data show that the effects of leptin on chloride homeostasis in vitro are restricted to a narrowed developmental window.

## Discussion

Besides its key role in regulating energy balance, leptin exerts many other important developmental and physiological functions throughout life [1, 2, 4, 7, 38]. In the present study, we show that leptin acts directly on newborn rat hippocampal neurons to control the chloride homeostasis and the strength of GABAergic inhibition in vitro. We further show that the effects of leptin rely on the control of the activity of the K/Cl cotransporter KCC2 and are present during a restricted developmental window. The present study complements previous reports of leptin modulating GABAergic synaptic transmission in the developing rat hippocampus in vitro [12, 29] and extends our previous report of leptin controlling the ontogenesis of functional GABAergic inhibition in the developing mice hippocampus in vivo [13].

Our data demonstrate that bath applied leptin regulates the activity of KCC2 in the developing rat hippocampus. We have shown that leptin treatment induces a depolarizing shift of E_GABA_ and increases the firing frequency of CA3 pyramidal neurons. Both effects were prevented by the selective KCC2 blocker VU0463271. How acute (20 min) application of leptin controls KCC2 activity is presently unknown. The ion transport activity of KCC2 depends on transcriptional factors (i.e. the protein abundance) as well as post-translational regulations by (de)phosphorylation of the protein [33, 34]. We previously showed that newborn leptin receptor deficient (*db/db*) mice showed an increased expression of KCC2 compared to their wild type littermates [13]. We also showed that chronic (24 h) treatment of rat hippocampal neuronal cultures with leptin decreased the amount of KCC2 and increased the phosphorylation of the threonine 906 and 1007 residues (Thr906/Thr1007) of KCC2 [13], known to decrease the membrane expression and activity of the transporter [39, 40]. In the presence study, the acute (20 min) application of leptin was unlikely to induce transcriptional modifications, and a post-translational regulation is the most expected mechanism to account for the reduced activity of KCC2.

Developmental changes in leptin’s actions and downstream signaling pathways have been reported in the hippocampus [29, 35, 37] and hypothalamus [36]. We found that the effects of an acute (20 min) leptin (100 nM) application on E_GABA_ and firing of CA3 pyramidal neurons are also developmentally regulated been observed at P5 but not at P2 and P10 on rat hippocampal slices. We previously reported that a chronic (24 h) leptin (100 nM)-treatment had no effect on immature rat hippocampal neurons (DIV6), when KCC2 activity is low, but led to a depolarizing shift of E_GABA_ in more mature cultures (DIV15), when GABA had shifted to hyperpolarized values [13]. Differences in experimental systems and/or treatment protocols are possible explanations for the difference in leptin’s action in neuronal cultures versus acute slices at a time when the developmental shift of GABAergic responses had occurred (i.e. at DIV15 and P10 respectively). However, our observation that an acute application of leptin (100 nM, 20 min) induced a depolarizing shift of E_GABA_ in DIV15 neuronal cultures (unpublished observation) strongly suggest that differences in experimental system is the most likely explanation.

Different mechanisms, including a developmentally regulated expression of the leptin receptors as well as downstream signaling pathways and/or effectors could account for the developmental changes in leptin’s actions observed in hippocampal slices. The former hypothesis is unlikely since both molecular [8, 10] and functional [11, 12] studies revealed the presence of functional leptin receptors in the newborn rodent hippocampus. Accordingly, real-time qRT-PCR revealed the presence of Leptin receptor transcript in rat hippocampi at P2, P5 and P10 (unpublished observation). The latter hypothesis could be considered even if the downstream pathway linking leptin and the activity of KCC2 remains to be elucidated. The With No lysine family of serine/threonine kinase (WNK)-dependent phosphorylation of the Thr906/Thr1007 residues of KCC2 is a key player in the regulation of chloride homeostasis during development [39, 40]. We previously obtained evidence that a chronic leptin-treatment (24 h) promotes the phosphorylation of the Thr906/Thr1007 residues of KCC2 via a WNK-dependent pathway on hippocampal neuronal cultures [13]. Developmental changes in WNK signaling and WNK-dependent control of chloride homeostasis have been observed both in vitro and in vivo in cortical and hippocampal neurons [39, 40]. Moreover, Thr906/Thr1007 residues becomes progressively dephosphorylated during neuronal development [40, 41]. Thus, the high level of endogenous Thr906/Thr1007 phosphorylated KCC2 at birth and the absence of KCC2-dependent control of chloride homeostasis by endogenous WNK in mature neurons are possible explanations for the restricted effects of leptin.

A variety of factors control the activity of KCC2 and/or NKCC1 (for review see [42]). Among this array of factors, Neurturin, BDNF and oxytocin also displayed specific age-dependent actions on KCC2. Neurturin promotes the activity of KCC2 in hippocampal neuronal cultures [43]. This facilitatory effect peaked at DIV11 and declined with neuronal culture maturation, likely as a consequence of corresponding receptors expression. The effect of BDNF on KCC2 encompasses up regulation in immatures neuronal cultures [44] and down regulation in matures neuronal cultures [45] as a consequence of a change in the BDNF-receptor activated pathway [45]. Finally, oxytocin increases the membrane expression/stabilization of KCC2 during a very narrow time window (i.e. DIV3 and DIV4) in hippocampal neuronal cultures, but induced no significant change at DIV5 and a reduction at DIV6, most likely as a developmental change in oxytocin receptor activated pathway [46].

We have shown that an acute elevated concentration (i.e. 100 nM, but not 20 nM) of leptin affects the chloride homeostasis of the CA3 pyramidal neurons on newborn rat hippocampal slices. Likewise, in a previous study we reported that chronic hyperleptinemia, induced in vivo by daily sub-cutaneous injections of leptin from P2 to P10, delayed the emergence of functional GABAergic inhibition in the newborn mice hippocampus [13], while the same injections from P20 to P30 has no effect on GABAergic inhibition (unpublished observation). Elevated circulating leptin levels have been observed in patient with neurodevelopmental disorders such as Autistic spectrum disorder (ASD) and Rett syndrome [15, 16, 18, 46, 47] and in animal models of the diseases [48, 49]. Moreover, accumulating evidence indicate that impaired chloride homeostasis is a common feature of numerous neurological disorders associated with impairments in hippocampal-dependent cognitive processes [30–32]. Although translating animal research to the human situation is difficult, the developmental window of the actions of elevated leptin levels on GABAergic inhibition in vivo and in vitro is consistent with a possible role of elevated leptin levels in neurodevelopmental disorders. Targeting the leptin signaling pathway may therefore have therapeutic potential in neurological and neuropsychiatric disorders.

## Data Availability

The data sets generated for this study are available from the corresponding author upon reasonable request.

## Abbreviations

APV: 5-Amino-phosphono-valeric acid
ASD: Autistic spectrum disorder
*db/db* mice: Leptin-receptor deficient mice
BDNT: Brain-derived neurotrophic factor
eGABA_A_-PSCs: Evoked GABA_A_ receptor-mediated postsynaptic currents
DIV: Days in vitro
GABA: γ-Aminobutyric acid
KCC2: K^+^-Cl^−^ cotransporter
NBQX: 1,2,3,4-Tetrahydro-6-nitro-2,3-dioxo-benzo[f]quinoxaline-7-sulfonamide
NKCC1: Na^+^-K^+^-2Cl^−^ co-transporter
*ob/ob* mice: Leptin deficient mice
Thr906/Thr1007: Threonine 906 and 1007 residues
WNK: With no lysine family of serine/threonine kinase
